# Importance of the Occipitoaxial Angle and Posterior Occipitocervical Angle in Occipitocervical Fusion

**DOI:** 10.1111/os.12553

**Published:** 2019-11-19

**Authors:** Chao Tang, Guang Zhou Li, Ye Hui Liao, Qiang Tang, Fei Ma, Qing Wang, De Jun Zhong

**Affiliations:** ^1^ Department of Spine Surgery Affiliated Hospital of Southwest Medical University Luzhou China

**Keywords:** Clinical efficacy, Lower cervical curvature, Occipito‐C2 angle, Occipitocervical fusion, Posterior occipitocervical angle

## Abstract

**Objective:**

To observe the effects of occipitoaxial angle (O‐C2 angle, OC2A) and posterior occipitocervical angle (POCA) selection on postoperative clinical efficacy and lower cervical curvature in patients with acute acquired atlantoaxial dislocation after occipitocervical fusion (OCF).

**Methods:**

A total of 150 healthy subjects without cervical disease (healthy group) were randomly selected based on gender and age. Three spine surgeons measured the OC2A and POCA of the healthy group and averaged the values. A total of 30 patients with an average age of 51.0 years (range, 18–70 years; 16 male and 14 female) with trauma or rheumatoid arthritis (disease group) who underwent occipitocervical fusion (OCF) for atlantoaxial dislocation between January 2012 and June 2016 were reviewed. OC2A, POCA, and cervical spinal angle (CSA) were measured postoperative/soon after surgery and ambulation, and at the final follow‐up visit. The preoperative and final follow‐up visual analog scale (VAS), Japanese orthopedics association score (JOA), neck disability index (NDI), and dCSA (change of CSA from postoperative/soon after surgery and ambulation to final follow‐up) were recorded.

**Results:**

The values of OC2A and POCA in 150 healthy subjects were 14.5° ± 3.7° and 108.2° ± 8.1°, respectively, and the 95% confidence interval (CI) were 7.2°–21.8° and 92.3°–124.0°, respectively. There was a negative correlation between OC2A and POCA (r = −0.386, *P* < 0.001). There were 18 patients (group one) of ideal OC2A and POCA (both within 95% CI of the healthy group) postoperative/soon after surgery and ambulation with a mean follow‐up time of 26.3 ± 20.9 months in disease group. The remaining patients (group two) with a mean follow‐up time of 31.3 ± 21.3 months. There was no statistically significant difference in the baseline data as well as pre‐operative outcomes, including VAS score, JOA score, and NDI between the two groups. Likewise, the post‐operative outcomes in final follow‐up, including VAS and JOA score, had no distinct difference in the two groups. However, NDI (11.0 ± 2.9) in group two at the final follow‐up was significantly higher than that in group one (7.0 ± 2.3) (*P* < 0.001). And group two showed statistically greater dCSA (5.9 ± 7.5°) than group one (−2.3° ± 6.2°) (*P* = 0.003).

**Conclusions:**

The negative correlation between OC2A and POCA plays an important role in maintaining the biodynamic balance of the occipital‐cervical region. OC2A and POCA should be controlled of a normal population in patients with acute acquired atlantoaxial dislocation during OCF, which can further improve the clinical efficacy and prevent loss of lower cervical curvature after surgery.

## Introduction

Occipitocervical fusion (OCF) is an effective surgical procedure for the treatment of occipitocervical instability caused by trauma, inflammation, congenital diseases, cancer, and iatrogenic factors^1^, ^2^, ^3^. As OCF has increasingly been studied, it has become clear that the improper choice of occipitocervical angles have led to postoperative poor fusion, neck stiffness and pain, limitation of neck activity, and other symptoms, even including severe dysphagia and dyspnea^4^, ^5^, ^6^. Long‐term follow‐up indicated that improper occipitocervical angles not only caused clinical dissatisfaction among patients but may also cause abnormalities of the lower cervical curvature and accelerated degeneration^5^. Therefore, the choice of occipitocervical angles is particularly important if an ideal occipitocervical angle is to be maintained.

Currently, there is no clear gold standard for establishing the ideal occipitocervical angle for OCF. Occipitoaxial angle (O‐C2 angle, OC2A), owing to its simple measurement method and high reliability, has being one of the most common methods for researchers to measure the occipitocervical angle. Many researchers have paid special attention to the clinical effects of the OC2A in OCF after surgery and its relation to long‐term lower cervical degeneration^7^, ^8^, ^9^, ^10^. However, intraoperative adjustment of the OC2A curvature through the use of a contoured rod is complicated and affected by the quality of intraoperative fluoroscopy^7^. Riel *et al*. have firstly proposed the application of posterior occipitocervical angle (POCA) as this method is highly correlated with the measurement methods of the earlier literature, with good consistency among the observers^11^. This method is believed to facilitate the design and measurement of the implants used in OCF. Maulucci *et al*. found that when the POCA is too high, it leads to increased biological stress on adjacent areas, which can require revision surgery and may be related to the incidence of postoperative dyspnea^12^.

However, none of the relevant literature has reported on the analysis of both OC2A and POCA selection in OCF. Therefore, the main goals of this study are to: (i) measure the OC2A and POCA in 150 healthy adults and provide a normal reference range (95% CI) for OC2A and POCA; (ii) explore the effects of OC2A and POCA on the clinical efficacy and lower cervical curvature in patients with acquired atlantoaxial dislocation for trauma or rheumatoid arthritis after OCF; and (iii) summarize the existing limitations and the possible direction for further research.

## Materials and Methods

### *The Inclusion and Exclusion Criteria in Healthy Group*

Inclusion criteria: (i) the subjects were aged between 20 and 70 years and with no cervical deformity or other cervical related diseases (e.g. cervical spondylotic radiculopathy or myelopathy, etc.); (ii) x‐ray examination was performed in a standing neutral position; and (iii) the structure of hard palate, occipital bone and cervical vertebra can be clearly displayed on x‐ray, and OC2A and POCA can be accurately measured.

**Exclusion criteria:** (i) straightening cervical curvature or kyphosis; (ii) combined with thoracic and/or lumbar diseases affecting sagittal sequence of the spine.

One hundred and fifty healthy subjects (healthy group) were screened randomly in the physical examination center according to the inclusion and exclusion criteria, and were divided into male and female groups of 75 members each. In terms of age, there were five groups of 15 members each, ranging in age from the second to the seventh decade.

### *The Inclusion and Exclusion Criteria in Disease Group*

Inclusion criteria: (i) the subjects were aged between 18 and 78 years; (ii) acquired cranio‐cervical diseases with atlantoaxial dislocation (AAD) (e.g. trauma or rheumatoid arthritis, etc.); (iii) the patients with a surgery of OCF, and cervical pedicle screws and/or lateral mass screws were performed during the surgery; (iv) the postoperative follow‐up time of patients was longer than 6 months, and had complete clinical follow‐up data, including VAS, JOA, NDI, and CSA; and (v) all patients signed informed consent at final follow‐up.

Exclusion criteria: (i) degenerative disease of lower cervical spine with related clinical symptoms; (ii) combined with basilar invagination or chiari malformation; (iii) history of lower cervical spine surgery; (iv) the grade of American Spinal Injury Association (ASIA) was A or B at final follow‐up; (v) combined with other spinal deformities or spinal sagittal imbalance.

A total of 30 patients (disease group) who underwent instrumental OCF for atlantoaxial dislocation in our hospital between January 2012 and June 2016 were reviewed. Our hospital's institutional review board approved the study.

### *Patient Information in Disease Group*

The disease group included: 16 males (53.3%, 16/30) and 14 females (46.7%, 14/30) aged 51.0 ± 15.8 years (18–78 years), with an average follow‐up time of 28.3 ± 20.8 months (6–78 months). The diagnoses included atlantoaxial fracture and dislocation (93.3%, 28/30), rheumatoid atlantoaxial dislocation (6.3%, 2/30). All patients received occipitocervical fixation due to severe Jefferson fracture and/or C1 lateral mass fracture resulted in difficulty in atlantoaxial fixation.

### *Surgical Procedure in Retrospective Series*

#### *Anesthesia and Surgical Position*

All patients underwent intraoperative distraction and fixation procedure as described previously^13^. After induction of general anesthesia, the patient was placed in the prone position with the head fixed in a Mayfield head holder. The neck was positioned in a slightly flexed position, allowing for better surgical exposure of the cranio‐vertebral junction using the posterior approach.

#### *Approach and Exposure*

*Via* a posterior midline incision, surgical exposure was accomplished from the occiput to the cervical spinous process, bilateral laminae and articular processes. Selection of the exposure range of cervical spinous process is made according to occipitocervical fixation segment. Exposure range from occipital bone to C2 occurred in 25 patients, four patients were accomplished from the occiput to the C3 spinous process due to the difficulty of C2 pedicle or lateral mass screw placement with high‐riding vertebral artery, and one patient's exposure to C4 spinous process owing to laminar and bilateral lateral mass fracture of C3.

#### *Fixation and Reduction*

One pair of pedicle or lateral mass screw was inserted into C2 or C3 according to the exposure range. Two pairs of pedicle screws were implanted into bilateral pedicles of C3 and C4 in one patient. The appropriate length of titanium rod was intercepted and pre‐bended according to the angle of occipitocervical junction. Three screw connectors were placed at the head end, and three pairs of occipital nails were placed at the scales of occipital bone through bilateral titanium rod connectors.

The reduction of AAD was per‐formed *via* two steps. First, the facet joint capsule between atlas and axis was opened, and the cartilage over the joint was removed using a fine drill. Second, cervical pedicle or lateral mass screws were tightly fastened, while occipital screws were only loosely tightened at this step. Distraction between the occipito‐cervical junction of the rod and occipital screws was performed, and the occipital screws were subsequently tightened. Consequently, the C2 was dragged inferanteriorly, which enabled horizontal and partial vertical reduction. Motor‐evoked potential monitoring following transcranial electrical stimulation (MEP) was performed during surgery.

#### *Reconstruction and Fusion*

Skeletal knife carefully removed cortex and scales between titanium rods in the posterior middle of occipital bone. Electric grinding drill grinded the posterior arch of atlas and the bottom of C2‐3 spinous process, bilateral lamina and articular process to prepare bone graft bed. Granular autogenous cancellous bone was laid on the surface of the bone graft bed. Proper compression ensured close contact with the implant bed.

### *Radiological Analysis*

All subjects in healthy and disease groups underwent lateral cervical radiography using a DR device (Ziehm Solo) with fluoroscopy settings of 0.2 to 16 mA and 40 to 110 kV and a distance of 150 cm between the film and the x‐ray tube. The subjects were in a neutral position looking straight ahead; cervical lateral plain radiographs were taken with the fifth cervical vertebra as the center. Measurements of OC2A, POCA and CSA were performed on the images of the cervical lateral x‐rays in the Centricity PACS system (version 2.0, GE Healthcare, Milwaukee, USA).

#### *Measurement Methods of OC2A, POCA, and CSA*

**Figure 1:**
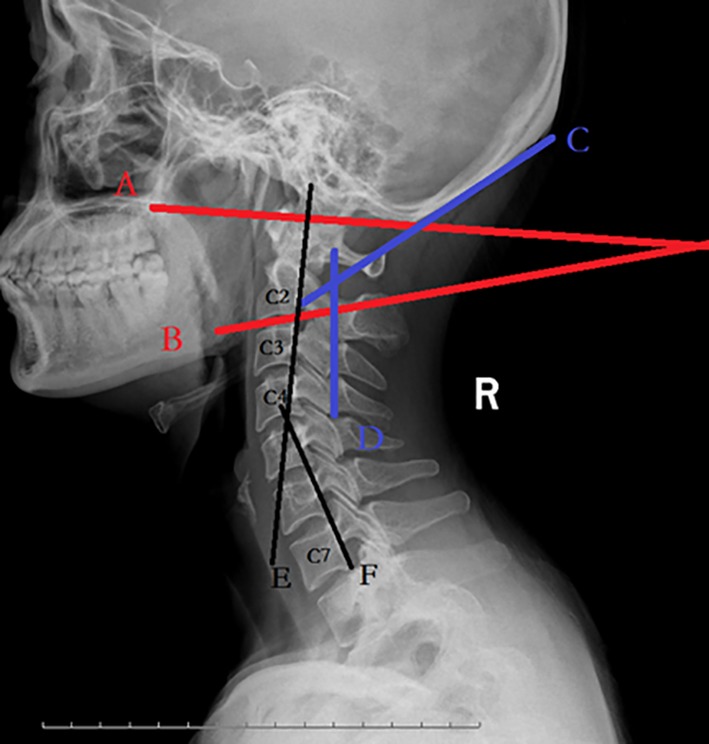
X‐ray lateral cervical spine in neutral position showing various angles. Occipitoaxial angle (OC2A): Angle between McGregor line (A line) and the line (B line) tangential to the inferior aspect of the axis. Posterior occipitocervical angle (POCA): Angle formed by the intersection of a line (C line) drawn tangential to the flat posterior aspect of the occiput between the foramen magnum and occipital protuberance and the line (D line) determined by the posterior aspect of the third and fourth cervical facets. Cervical spine angle (CSA): Angle between the posterior aspect of vertebral bodies of C2 (E line) and C7 (F line).

The OC2A defined by the angle between McGregor line and the line tangential to the inferior aspect of the axis. The POCA formed by the intersection of a line drawn tangential to the flat posterior aspect of the occiput between the foramen magnum and occipital protuberance and the line determined by the posterior aspect of the third and fourth cervical facets. The CSA was measured according to Gore *et al*.^8^ and was defined as the acute angle between the lines parallel to the posterior edges of the C2 and C7 vertebra bodies (Fig. 1).

#### *Contents of Imaging Measurement in Healthy Group and Disease Group*

OC2A and POCA in the healthy group were independently measured by three spine surgeons, and the average value was used for analysis. OC2A, POCA, and CSA were measured in the disease group postoperative/soon after surgery and ambulation, and at final follow‐up. The change of CSA (dCSA) from postoperative/soon after surgery and ambulation to final follow‐up after surgery was calculated.

$$\text{dCSA}\left({}^{\circ}\right)=\left(\mathrm{CSA}\;\text{of postoperative}/\text{soon after surgery and ambulation}\right)-\left(\mathrm{CSA}\;\text{of final follow}-\mathrm{up}\;\text{after surgery}\right)$$

### *Evaluation of Clinical Efficacy in Disease Group*

#### *Visual Analogue Scale*

The visual analogue scale (VAS) was performed to estimate the pain level of the patients. The VAS pain scoring standard (scores from 0 to 10) was as follows: 0 = painless; less than 3 = mild pain that the patient could endure; 4–6 = patient was in pain that could be endured and was able to sleep; and 7–10 = patient had intense pain and was unable to tolerate the pain.

#### *Japanese Orthopaedics Association Score*

Japanese Orthopaedics Association score (JOA) was evaluated by 1994 JOA Score system^14^, which consists of six categories: motor function of upper extremity and lower extremity; sensory function of upper extremity, trunk, and lower extremity; and function of the bladder.

#### *Neck Disability Index*

Neck disability index (NDI) is a self‐rated disability questionnaire developed for patients with neck pain. It consists of 10 items: pain intensity, personal care, lifting, reading, headaches, concentration, work, driving, sleeping, and leisure activities. Each item is scored from 0–5, no disability to total disability, with the maximum score being 50.

VAS, JOA score, and NDI were recorded before operation and at final follow‐up after operation.

### *Statistical Analysis*

SPSS software version 17.0 (SPSS Inc., Chicago, IL) was used for statistical analysis. A difference analysis was performed using the independent/paired ‐sample *t* test, the differences of OC2A and POCA values among all age groups in healthy group were compared by one‐way ANOVA analysis of variance. χ^2^ test was used for categorical variables. Pearson correlation coefficient was used for OC2A and POCA correlation analysis, *P* < 0.05 was considered statistically significant.

## Results

### *OC2A and POCA Measurements of 150 Healthy Subjects in the Healthy Group*

The OC2A and POCA of 150 healthy subjects were normal distribution, and the mean values were 14.5° ± 3.7°and 108.2° ± 7.8°, respectively. The 95% confidence intervals (CI) of OC2A was 7.2°–21.8°, and the CI of POCA was 92.3°–124.0°.

#### *Differences in OC2A and POCA between males and females in the healthy group*

The values of OC2A were 14.8° ± 3.1° and 14.2° ± 4.3° in males and females, respectively, and the values of POCA were 108.3° ± 7.8° and 108.0 ± 8.4° in males and females in the healthy group. There were no significant differences in OC2A or POCA between males and females (*P* > 0.05) (Table 1).

**Table 1:** Gender and the OC2A and POCA values in healthy group

	Sex	N	Mean value (°)	SD (°)	Standard error of mean (°)	*P*
CO2A	Male	75	14.7664	3.08554	0.89709	0.861
	Female	75	14.2177	4.26565	0.97058	
POCA	Male	75	108.2747	7.7690	0.49256	0.386
	Female	75	108.0430	8.40548	0.35629	

OC2A, occipitoaxial angle; POCA, posterior occipitocervical angle.

### *Changes of OC2A and POCA with Age in the Healthy Group*

The value of OC2A in the 20–29 years old group (n = 30, 15 of males and female each) was 15.3° ± 4.1°, 15.2° ± 3.0° in 30–39 years old group (n = 30, 15 of males and female each), 13.9° ± 3.8° in 40–49 years old group (n = 30, 15 of males and female each), 13.2° ± 3.9° in 50–59 years old group (n = 30, 15 of males and female each), and 14.7° ± 3.4° in 60–69 years old group (n = 30, 15 of males and female each). There was no significant difference in OC2A among all age groups (*P* > 0.05).

The value of POCA in 20–29 years old group was 110.5° ± 7.3°, 108.4° ± 7.9° in 30–39 years old group, 109.5° ± 3.4° in 40–49 years old group, 108.6° ± 3.4° in 50–59 years old group, and 103.8° ± 3.4° in 60–69 years old group. The values of POCA in healthy subjects over 60 years old was significantly lower than that in the other age groups (*P* < 0.05) (Table 2).

**Table 2:** Age and the OC2A and POCA values in healthy group

	20 + (n = 30†)	30 + (n = 30†)	40 + (n = 30†)	50 + (n = 30†)	60 + (n = 30†)	*F value*	*P value*
OC2A (°, Mean ± SD)	15.3 ± 4.1	15.2 ± 3.0	13.9 ± 3.8	13.2 ± 3.9	14.7 ± 3.4	1.836	0.125
POCA (°, Mean ± SD)	110.5 ± 7.3	108.4 ± 7.9	109.5 ± 8.2	108.6 ± 7.0	103.8 ± 8.6*	3.221	0.014*

OC2A, occipitoaxial angle; POCA, posterior occipitocervical angle.

* Statistically significant and the POCA over 60‐year‐old was smaller than other age groups.

† Combination of male and female in the same age group.

### *Analysis of the Correlation Between OCA and POCA*

**Figure 2:**
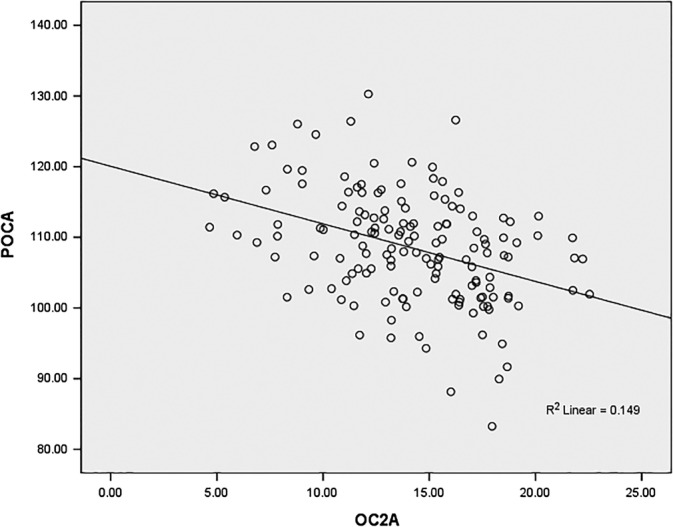
Relationship between the OC2A and POCA. There was a significant negative correlation between OC2A and POCA in healthy individuals (r = −0.386, *P* < 0.001).

Pearson correlation analysis showed that OC2A was negatively correlated with POCA in the healthy group (r = −0.386, *P* < 0.001) (Fig. 2).

### *Selection of OC2A and POCA Fixed States in Patients with OCF*

The OC2A and POCA of 30 patients were 19.5° ± 5.8° and 109.3° ± 8.7° at the postoperative/soon after surgery and ambulation, respectively. And the values of OC2A and POCA were 18.9° ± 5.6° and 111.2° ± 8.1° at final follow‐up time. There was no significant difference in OC2A or POCA between postoperative/soon after surgery and ambulation and final follow‐up in the disease group (*P* > 0.05).

Of 30 patients with OCF in the disease group, 18 patients (60%) with an ideal OC2A and POCA (both within 95% CI of the healthy group) postoperative/soon after surgery and ambulation were divided into group one; 12 patients (40%) with a non‐ideal OC2A and POCA postoperative/soon after surgery and ambulation were divided into group two.

### *The Results of Clinical Efficacy in the Disease Group*

The clinical and demographic data for the patients with OCF is summarized in Table 3.

**Table 3:** Data of the 30 patients with occipitocervical fusion case

Case	Age/gender	Diagnosis	Fixation level	Ideal/non‐ideal angle of OC2A/POCA at post‐o(P‐S)	Pre‐O			POST‐O(P‐S)			POST‐O(F‐F)						F/U(m)	Note
					VAS	JOA	NDI	OC2A	POCA	CSA	OC2A	POCA	CSA	VAS	JOA	NDI		
1	60/F	AAFD	O‐C2	Y	5	12	20	10.2	112.5	29.5	9.3	118.2	32.1	1	16	6	6	LMF
2	18/M	AAFD	O‐C2	Y	7	14	19	11.9	107.3	3.5	7.1	120	19.8	2	16	6	40	LMF
3	72/M	AAFD	O‐C3	Y	7	12	21	12.6	109.3	27.2	11.3	111.4	30	3	15	8	16	JF/LMF/HVA
4	78/M	AAFD	O‐C2	Y	6	14	16	11.4	112.5	−2.5	13.6	116.4	3.7	2	16	6	9	LMF
5	34/F	AAFD	O‐C2	Y	7	10	28	16.5	112.8	17.4	17.4	110.4	18	2	15	14	13	LMF
6	65/M	AAFD	O‐C2	Y	8	12	32	20.2	106.9	−4	22.1	119.5	−6	2	16	9	13	JF
7	61/M	AAFD	O‐C2	Y	6	14	18	16.3	109.9	21.8	13.5	117	23.4	1	16	6	78	HVA
8	62/F	AAFD	O‐C2	Y	6	14	18	18.4	113.4	15.9	18.6	108	22	2	16	6	6	LMF
9	57/F	AAFD	O‐C2	Y	7	14	20	10.2	115.5	20.7	12	120.1	21.9	1	16	7	24	JF/HVA
10	64/F	AAFD	O‐C2	Y	6	13	23	12.8	118.5	26.9	11.5	121.6	32.8	2	16	5	26	JF
11	18/M	AAFD	O‐C2	Y	4	15	14	16.4	105.6	15	16	93.4	16.7	2	16	8	20	JF
12	50/F	RAD	O‐C2	Y	8	14	18	21.7	106.9	21.3	26.3	114.1	36.7	2	16	9	49	‐
13	52/F	AAFD	O‐C4	Y	7	14	22	13.2	122.8	11.8	14.8	119.6	13.9	2	16	9	16	LMF/HVA/LF
14	45/M	AAFD	O‐C2	Y	8	12	27	14.1	105.1	18.4	12.3	109.7	21.4	3	16	7	25	LMF
15	68/M	AAFD	O‐C2	Y	6	15	13	11.2	109.5	−2.1	9.3	112	7.1	1	16	5	72	JF
16	54/F	AAFD	O‐C2	Y	6	14	15	11.2	118.4	21.3	8.5	117.7	24.9	2	16	6	24	LMF
17	46/M	AAFD	O‐C2	Y	6	14	17	14.6	105.2	16.5	16.9	110.3	14.7	2	16	5	12	JF
18	63/M	AAFD	O‐C2	Y	6	15	11	11.2	116.8	31.5	9.8	120.4	29.5	2	16	4	24	JF/LMF
19	62/M	AAFD	O‐C3	N	6	14	17	29.5	107.3	3.2	27.1	104.7	−13.9	3	15	7	12	LMF/HVA
20	51/F	AAFD	O‐C2	N	6	14	16	23.5	98.1	15	22.1	97.5	9.9	1	16	11	6	HVA
21	43/F	AAFD	O‐C2	N	7	13	19	22.6	106.9	18.4	20.9	114.7	9.5	3	15	7	56	JF
22	61/M	AAFD	O‐C2	N	6	14	16	22.4	109.5	19.8	24.5	104.3	26.4	2	15	10	18	LMF
23	45/F	AAFD	O‐C2	N	7	15	18	31	104.8	−3.5	28.8	102.1	−8.8	3	16	11	27	LMF/HVA
24	31/F	AAFD	O‐C3	N	6	12	21	26.3	98.5	15.4	24.1	103	6.5	2	15	15	52	LMF
25	70/F	RAD	O‐C3	N	5	12	28	19.8	129.5	5.9	15.7	131.6	−8.9	3	15	14	24	HVA
26	42/M	AAFD	O‐C2	N	7	11	26	27.1	101.1	4.8	23.9	97.1	−5.9	3	15	16	48	LMF
27	29/M	AAFD	O‐C2	N	7	15	16	30.6	100	15.1	31.6	96.5	−19.4	2	16	11	36	HVA
28	42/M	AAFD	O‐C2	N	6	13	19	28.1	109.1	−5.2	21.6	114.6	3.1	1	15	8	16	LMF
29	60/M	AAFD	O‐C2	N	5	13	18	23.8	95.4	19.2	16.8	108.4	15.2	1	16	12	72	LMF
30	26/F	AAFD	O‐C2	N	7	14	17	6.5	128.1	2.3	7.8	125.6	−4.8	2	16	10	9	LMF

OC2A, occipitoaxial angle; POCA, posterior occipitocervical angle; CSA, cervical spinal angle; VAS, visual analog scale; JOA, Japanese orthopedics association score; NDI, neck disability index; Pre‐O, pre‐operation; Post‐O(P‐S), postoperative/soon after surgery and ambulation; Post‐O(F‐F), post‐operative final follow‐up; RAD, rheumatoid atlantoaxial; AAFD, atlantoaxial fracture and dislocation; JF, Jefferson fracture; LMF, lateral mass fracture; HVA, high‐riding vertebral artery; LF, laminar fracture.

The values of VAS, JOA, NDI in group one and group two were significantly improved at the final follow‐up after surgery compared with pre‐operation (*P* < 0.05) (Table 4).

**Table 4:** Comparative summary of patients with group 1 and group 2

	Group 1	Group 2	P value
Case number	18	12	0.529‡
Male	10	6	
Female	8	6	
Mean age (yrs)	53.5 ± 16.4	46.8 ± 14.2	0.261§
Follow‐Up (month)	26.3 ± 20.9	31.3 ± 21.3	0.524§
OC2A			
Post‐O (°P‐S)	14.3 ± 3.9	24.3 ± 6.6	<0.001§ ^,^ †
Post‐O (°F‐F)	13.9 ± 5.0	22.1 ± 6.4	0.001§ ^,^ †
POCA			
Post‐O (°P‐S)	111.6 ± 5.2	107.4 ± 11.0	0.166§
Post‐O (°F‐F)	114.4 ± 6.8	108.3 ± 11.3	0.075§
VAS			
Pre‐O	6.4 ± 1.0	6.3 ± 0.8	0.583§
Post‐O (°F‐F)	1.9 ± 0.6** ^,^ †	2.2 ± 0.8** ^,^ †	0.291§
JOA			
Pre‐O	13.4 ± 1.3	13.3 ± 1.2	0.820§
Post‐O (°F‐F)	15.9 ± 0.3** ^,^ †	15.4 ± 0.5** ^,^ †	0.072§
NDI			
Pre‐O	19.6 ± 5.4	19.3 ± 3.9	0.868
Post‐O (°F‐F)	7.0 ± 2.3** ^,^ †	11.0 ± 2.9** ^,^ †	<0.001§ ^,^ †
dCSA (°)	−2.3 ± 6.2	5.9 ± 7.5	0.003§ ^,^ *

dCSA = (CSA of postoperative/soon after surgery and ambulation)‐(CSA of final follow‐up after surgery).

OC2A, occipitoaxial angle; POCA, posterior occipitocervical angle; CSA, cervical spinal angle; VAS, visual analog scale; JOA, Japanese orthopedics association score; NDI, neck disability index; Pre‐O, pre‐operation; Post‐O (P‐S), postoperative/soon after surgery and ambulation; Post‐O (F‐F), post‐operative final follow‐up.

* Statistically significant between group 1 and group 2.

† Statistically significant between pre‐operation and post‐operative final follow‐up.

‡ Fisher's exact test.

§ Independent‐samples *t* test.

** Paired‐Samples *t* test.

The values of VAS and JOA were 1.9 ± 0.6 and 15.9 ± 0.3 in group one at the final follow‐up after surgery, respectively, 2.2 ± 0.8 and 15.4 ± 0.5 of VAS and JOA were recorded in group two. There was no significant difference in VAS and JOA between group one and group two at the final follow‐up (*P* > 0.05) (Table 4). But the NDI of the final follow‐up in group two (11.0 ± 2.9) was significantly higher than that in group one (7.0 ± 2.3) (*P* < 0.05).

### *Change of the Lower Cervical Curvature in the Disease Group After OCF*

The CSA of group one was 16.1 ± 10.9 postoperative/soon after surgery and ambulation, and 20.1 ± 10.9 at final follow‐up after surgery. CSA of 9.2 ± 9.0 and 4.7 ± 8.3 were measured in group two postoperative/soon after surgery and ambulation and final follow‐up time, respectively. The dCSA from group one (−2.3° ± 6.2°) was significantly lower than that from group two (5.9° ± 7.5°) (*P* < 0.05) (Table 4).

## Discussion

In 1910, Pilcher firstly reported the treatment of AAD by means of occipitocervical fixation^15^. Kraus *et al*. reported that in patients with rheumatoid arthritis, the incidence of postoperative subaxial subluxation for OCF was 36%^16^. Some researchers considered the occurrence of subaxial subluxation to be a natural course accompanied by degeneration change of joints and disc^17^. But the subaxial subluxation development within 1 year after surgery in most cases, which was too early to have been caused by a natural degeneration process, we considered. Whereas, in the current study, the findings indicate a significant association between the position of the occipital bone and the axis in the effect of postoperative lower cervical degeneration and clinical efficacy in OCF.

In OCF, caution should be paid to an ideal fixation angle between the craniocervical junction, becasue a non‐ideal angle can affect the position of the occipital bone and the axis, which may lead to dysphagia and respiratory disturbance and subaxial lordosis in long‐term follow up. OCA, as the most common measurement method for researchers to measure the occipitocervical angle, has already been concerned about its effects in subaxial degeneration and clinical efficacy after OCF. In 2001, Matsunaga *et al*. measured 240 healthy volunteers (120 males and 120 females) and found that OC2A were higher in females than in males in all age groups and that OC2A in males and females were gradually decreased after age 40^4^. In 2006, Sherekar *et al*. measured OC2A in 518 asymptomatic volunteers (261 male and 257 female) with OC2A values of 14.66° ± 9.5° in males and 15.59° ± 8.26° in females and no significant difference in OC2A between males and females^18^. We measured the OC2A in 150 healthy adults and found that the value was 14.5° ± 3.7° (4.4°–25.5°) and the 95% CI was 7.2°–21.8°. Although OC2A in males was slightly higher than that in females in this study, we also found that these values had no significant difference. In addition, there was no significant difference in OC2A among age groups in both males and females. Some researchers have concluded that the degeneration and lordosis of the lower cervical curvature decreased with further changes in the biomechanical environment of the upper cervical spine after OCF^4^, ^5^. The decrease of mid‐to lower‐cervical lordosis acts as a compensatory mechanism for lordotic correction between the occiput and C2; this compensatory alignment change occurred immediately after ambulation and was maintained for follow‐up. POCA, as a simple method for the measurement of occipitocervical angle, might be more useful in implant design and testing, and where precontouring of fusion implants could aid in achieving and reproducing optimal fusion position. Riel *et al*. measured POCA values in 30 healthy volunteers with a POCA value of 109.7° ± 5.7°, and 80% of POCA values ranging from 101 to 119°, but there was no further analysis of how POCA may be impacted by differences in age and sex^11^. Kunakornsawat measured POCA values of 107.9° ± 4.4° (94°–120°) in 326 volunteers^19^. Our results showed that the POCA of the healthy group was 108.2° ± 8.1° (78.4°–130.8°) and the 95% CI was 92.3°–124.0°. The POCA of males was slightly higher than that of females, but the difference was not statistically significant. Maulucci *et al*. considered that when the POCA is too high in OCF it leads to increased biological stress on adjacent areas, which can require implant failure and may be related to the incidence of postoperative dyspnea^12^.

**Figure 3:**
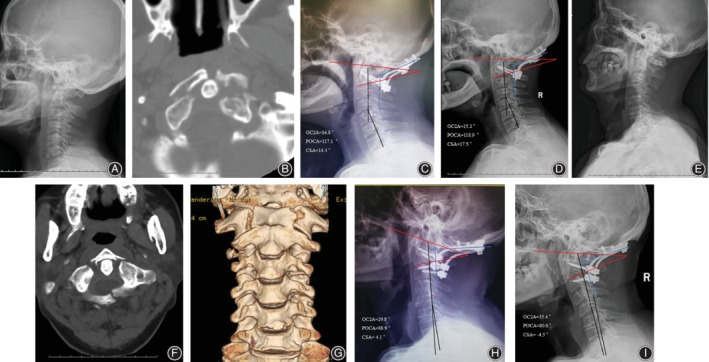
A 64 year old female with atlas fracture and atlantoaxial dislocation (A, B), who was established in an ideal angle of OC2A and POCA (both within 95% CI of the healthy population) during OCF (C). Cervical lateral radiograph showed a fairly good curvature of the lower cervical spine at 18 months follow‐up (D). On the contrary, a 62 year old male with Jefferson fracture (E, F, G), OC2A and POCA of the patient were established in a non‐ideal angle in OCF (H). The lower cervical spine of the patient had a kyphosis due to loss of the cervical curvature at 12 months after OCF (I).

OC2A and POCA, as key factors affecting the clinical efficacy and subaxial degeneration after OCF, maintain the biodynamic balance of the occipitocervical region together. Our study firstly analyzed the correlation between OC2A and POCA in healthy subjects, and the results showed that OC2A was negatively correlated with POCA (*r* = −0.386, *P* < 0.001). Previous studies reported in the literature focused more on the correlation analysis between the OC2A and lower cervical degeneration or changes in curvature^5^, ^18^. We concluded that OC2A, as an important occipitocervical angle of the occipitocervical junction, is not only related to the lower cervical spine but may also play an important fulcrum role in maintaining the balance of the occipitocervical region. We speculated, in the occipitocervical area, the OC2A compensation limits the downward and forward movement of the head to maintain looking straight ahead when the POCA is enlarged. In this study, 18 patients (group one) of ideal OC2A and POCA postoperative/soon after surgery and ambulation in disease group, The NDI of the final follow‐up in group one was significantly lower than that in group two. In our opinion, maintaining a normal reference range of occipitocervical angles would be more helpful for the recovery of the function position of the occipital bone and the axis in OCF. Too large or too small an occipitocervical angle can lead to long‐term stretching of the neck muscles or an increased concentration of facet joint stress, resulting in neck pain and other axial symptoms. Moreover, the dCSA in group one was significantly lower than that in the group two in our study. Guo *et al*. reported negative correlation between OC2A and CSA in asymptomatic population^9^. Passias *et al*. reported that OC2A and CSA negatively correlated preoperatively and postoperatively^10^. Those previous studies showed that the compensatory mechanism between occipito‐cervical region and subaxial cervical spine, and this compensatory alignment change, occurred immediately after ambulation and was maintained for follow up. In this study, there were 10 (10/12) patients with OC2A larger than 21.8° (the upper limit of normal in the reference range in the healthy group) in group two; we also found that there were patients with significant loss of lower cervical curvature in this group that in group one. We concluded that unbalanced biomechanical environment due to non‐ideal OC2A and POCA, increase compensatory mechanism between occipito‐cervical region and subaxial cervical spine and could affect the curvature of the lower cervical lordosis (Fig. 3).

**Figure 4:**
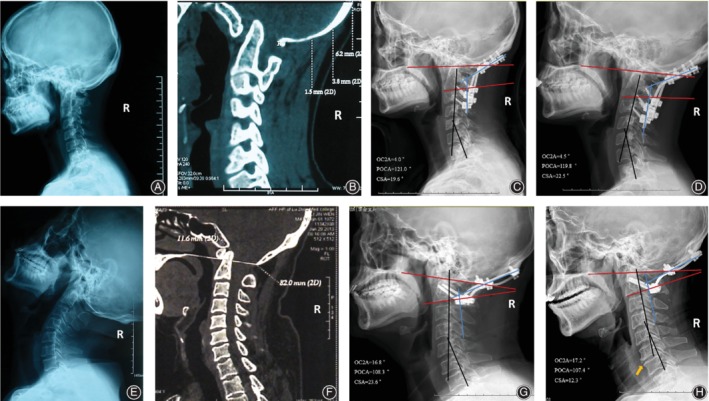
A 32 year old female with basilar invagination (BI) (A, B), OC2A and POCA of the patient were established in a non‐ideal angle in OCF (C). Follow‐up after surgery at 24 months showed that lower cervical curvature of the patient could be able to maintain better than that at postoperative/soon after surgery and ambulation (D). But another patient, a 41 year old male with BI (E, F), who had a severe loss of CSA and disc degeneration at 12 months follow‐up visit (H), established in an ideal reference range of his occipitocervical angle parameters during surgery (G).

In this study, is worth noting whether 95% CI of OC2A and POCA in healthy subjects is suitable for all occipito‐cervical region diseases in OCF? We found that the patients with basilar invagination and craniocervical malformation, with OC2A and POCA fixed at an ideal angle, had a severe loss of CSA at follow‐up visit after OCF. On the contrary, OC2A and POCA in patients with basilar invigilation fixed less than the lower limit of the ideal reference range during surgery, resulted in cervical curvature of the patients being better maintained at postoperative follow‐up (Fig. 4). Researchers believe that basilar invagination patients with cervical deformities, loss or destruction of atlantoaxial ring structure, atlanto‐occipital fusion, and C2–3 fusion will have a high position of the odontoid process of the axis. Atlantoaxial lateral joint deformities will result in the concentration of centralized atlantoaxial joint stress and increased tension on the atlantoaxial transverse and the ligamentous ligaments^20^, ^21^, ^22^, ^23^. But the occipitocervical region maintains biomechanical balance by changing the compensatory mechanism of occipitocervical angle. In our opinion, we think that occipitalcervical angles of basilar invagination patients were fixed on the reference range of normal population, may lead to imbalance of occipital cervical region, thereby increasing the stress concentration of lower cervical vertebra.

The limitations of this study are as follows. First, cervical screw placement methods include pedicle screw and lateral mass screw in this study. As reported in previous literature, there are differences in biomechanical stabilization between the two methods^24^, ^25^. Therefore, it is not clear whether there are differences in the effects of cervical degeneration. Second, in this study, we have found an interesting phenomenon that 95% CI of OC2A and POCA in healthy subjects may not be suitable for patients with craniocervical malformation, such as basilar invagination, but there was an absence of control groups of basilar invagination patients. In further studies, we will observe the effect of ideal normal range OC2A and POCA on the degeneration of lower cervical spine after OCF in BI patients by retrospective study.

### *Conclusions*

We concluded that the negative correlation between OC2A and POCA might play an important role in maintaining the biodynamic balance in the occipitocervical region. The OC2A and POCA should be controlled in the 95% CI reference range of normal population in patients with acute acquired cranio‐cervical diseases during OCF, which can further improve clinical efficacy and reduce the loss of long‐term cervical convex curvature after surgery.
